# Cell and Gene Therapy for Spine Regeneration: Mammalian Protein Production Platforms for Overproduction of Therapeutic Proteins and Growth Factors

**DOI:** 10.1016/j.nec.2019.08.015

**Published:** 2020-01

**Authors:** Ali Mobasheri, Stephen M. Richardson

**Affiliations:** a Department of Regenerative Medicine, State Research Institute Centre for Innovative Medicine, Santariskiu 5, Vilnius 08661, Lithuania; b Research Unit of Medical Imaging, Physics and Technology, Faculty of Medicine, University of Oulu, PO Box 5000, Oulu FI-90014, Finland; c Centre for Sport, Exercise and Osteoarthritis Research Versus Arthritis, Queen’s Medical Centre, Nottingham, UK; d King Abdulaziz University, Jeddah, Kingdom of Saudi Arabia; e Division of Cell Matrix Biology and Regenerative Medicine, School of Biological Sciences, Faculty of Biology, Medicine and Health, University of Manchester, Manchester Academic Health Sciences Centre, Oxford Road, Manchester, UK

**Keywords:** Low back pain (LBP), Spine degeneration, Cell therapy, Gene therapy, Mammalian protein production platforms, Protein packaging cell lines, Growth factors, Transforming growth factor-β (TGF-β), GDF6

## Abstract

This article provides an evidence-based personal perspective on the future of cell and gene therapy for degenerative diseases of the intervertebral disc. This paper focuses on how mammalian protein production platforms and transfected and irradiated protein packaging cell lines may be used as “cellular factories” for overproduction of therapeutic proteins and proanabolic growth factors, particularly in the context of regenerative therapies. This paper also speculates on future opportunities and challenges in this area of research and how new innovations in biotechnology affect cell and gene therapy for degenerative diseases.

## Key points

• Cell and gene therapy for degenerative diseases of the joints and the spine is a promising area of research with significant potential for clinical development.
• Advances in biotechnology are likely to have a positive impact on tissue engineering and regenerative treatments for the spine.
• However, researchers developing cell and gene therapies for the spine will need to accept the harsh reality that primary, aged, and senescent cells will possess feeble regenerative properties.
• Regenerative medicine and tissue engineering strategies for the spine should therefore consider the use of stem cells combined with mammalian protein production platforms to drive the production of therapeutic proteins and proanabolic growth factors.
• Protein production tools are essential for faster progress in cellular therapy.

## Introduction

Degenerative changes in the intervertebral disc (IVD) cause the loss of normal spine structure and function.^1^ IVD degeneration is not typically due to a specific injury but rather it is related to aging. It is possible that injuries can influence the long-term course of spine degeneration. However, spine degeneration is a biomechanically related continuum of molecular, biochemical, cellular, and anatomic alterations evolving over time, due to external insults, such as mechanical or metabolic injury.^2^ Human beings are living longer lives and expect to enjoy pain-free mobile lifestyles. Degenerative diseases of the joints and the spine are largely associated with aging, obesity, poor diets, and occupational factors. Although our ancestors have been around for about 6 million years, modern humans (*Homo sapiens*) only evolved about 200,000 years ago. Early humans did not live long enough to suffer from age-related musculoskeletal conditions, and degenerative diseases of the joints and the spine are thought to have been extremely rare in early humans, but this is probably because of an underrepresentation of older adults in the skeletal records of the ancient civilizations. Longer life expectancy and the strains, sprains, and overuse of the back over many decades result in a gradual IVD degeneration in the spine.^3^ According to the World Health Organization, low back pain (LBP) is a leading cause of disability across the world.^a^ LBP occurs in similar proportions in all cultures, interferes with quality of life and work performance, and is currently the most common reason for medical consultations globally. Although LBP has many causes, IVD degeneration has been shown to be an important underlying cause.^4^

**Fig. 1:**
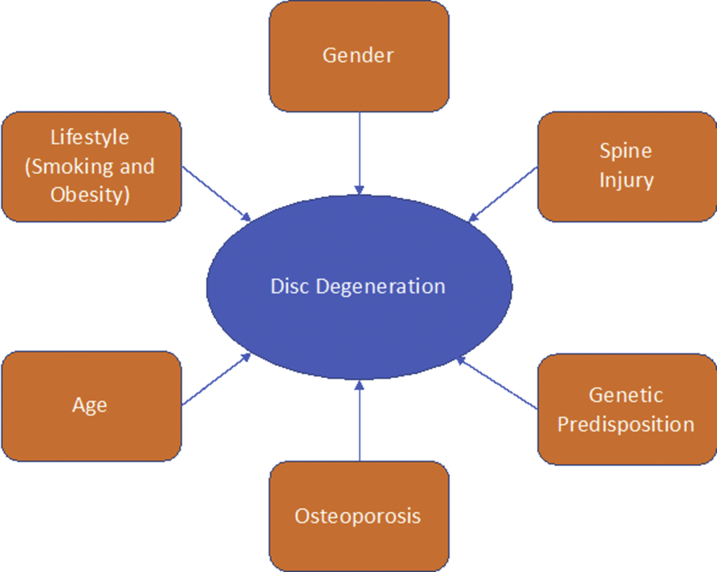
Risk factors for spine degeneration.

Despite the growing prevalence and burden of LBP, IVD, and spine degeneration, there are no effective cures. Degenerative changes in the spine are associated with biomechanical and metabolic alterations and it has been proposed that the degeneration is an adaptation, rather than a disease.^2^ It has also been proposed that in the absence of a cure for LBP, IVD, and spine degeneration, the only way to reduce the global burden of these conditions is developing earlier diagnostics, improving management regimes, and conceiving realistic long-term strategies for prevention. Diagnosis of all degenerative joint and spine conditions begins with radiography. However, MRI is increasingly used to image discs, nerves, and the spinal canal space. Computed tomography may be used to resolve inconsistencies between the MRI and the patient's symptoms. Disc studies, also known as discograms, may be ordered to determine if a patient's pain is being caused by a damaged spinal disc. Treatment depends on the type and severity of the patient’s condition. In most cases, nonsurgical treatment is all that is required. These treatments may include exercise to increase flexibility and muscle strength, braces, or medication. Pain medication and steroids may be administered via epidural injection. In extreme cases surgery may be required for herniated discs or spinal stenosis, particularly where there is radicular pain. The treatment applied is often for radiculopathy, that is, sciatica, or other nerve issues that cause loss of function, rather than for the LBP itself. In addition to age, gender, lifestyle, and genetic predisposition, other inciting risk factors for disc degeneration may include previous spine injuries or even osteoporosis (Fig. 1). This article focuses on IVD degeneration and how cell and gene therapy may be used for spine regeneration.

## The hallmarks of spine degeneration

All human diseases are characterized by “hallmarks,” which summarize the key biological alterations that occur in a particular disease. For example, cancer comprises 6 biological capabilities that are gradually acquired during the multistep development of human tumors.^5^, ^6^ In the case of spine degeneration, there are many similarities with the hallmarks of aging.^7^ The hallmarks of aging include genomic instability, telomere attrition, epigenetic alterations, loss of proteostasis, deregulated nutrient sensing, mitochondrial dysfunction, cellular senescence, stem cell exhaustion, and altered intercellular communication. Many of the hallmarks of aging are also seen in IVD degeneration and in articular cartilage in degenerative joint diseases such as osteoarthritis (OA).

## Cellular senescence

Senescence of the cells in the specialized tissues of the IVD is a normal part of aging^8^; however, cellular senescence has been shown to be accelerated in degeneration^9^ with a range of causes proposed.^10^ There is a gradual decline in cell number in the IVD with aging through increased apoptosis, and secondary necrosis reduces the cellularity of the tissue and its ability to repair and regenerate. However, there are reports that there is also increased cellularity in some areas of degenerate discs, with clusters of chondrocyte-like cells forming by cell proliferation in degenerating areas.^11^ Interestingly, regional chondrocyte hypocellularity and cloning is also seen in degenerating articular cartilage,^12^, ^13^ reminding us of the many similarities between IVD and cartilage, especially with regard to extracellular matrix (ECM) composition.

Changes in cellularity are important because they alter the nutritional status and metabolic substrate requirements of the disc and impact on the concentration gradients of nutrients, metabolites, and waste products.^11^ The normally avascular disc in the healthy adult can become increasingly vascularized and innervated with degeneration and disease. This may lead to an increased supply of oxygen and nutrients to the disc, altering the metabolism of the disc and the phenotype of its resident cells. Furthermore, vascularization and innervation can also introduce other cell types, including inflammatory cells, and a range of bioactive molecules such as proinflammatory cytokines and growth factors. The increased production and secretion of proinflammatory cytokines, particularly tumor necrosis factor α and interleukin 1 β, drives autophagic changes as well as cell death.^14^ The relationship between autophagy, apoptosis,^15^, ^16^ cell senescence,^17^, ^18^ and mitochondrial dysfunction has not been extensively explored in the aging and degenerating disc, and it has been suggested that all these mechanisms are implicated in spine degeneration. Senescent cells cannot divide and they promote the development of a senescence-associated secretory phenotype (SASP). Research at the intersection between the fields of oncology and immunology has demonstrated that the acquisition of SASP can turn senescent stromal fibroblasts into proinflammatory cells that have the ability to promote tumor progression.^19^ So what is the relevance of this phenomenon to degenerative diseases of the joints and the spine? SASP defines the ability of senescent cells to express and secrete a variety of extracellular modulators that includes cytokines, chemokines, proteases, growth factors, and bioactive lipids. The SASP secretome can mediate chronic inflammation and stimulate the growth and survival of aggressive and persistent cells with inflammatory potential.^20^ SASP may reduce the disc's ability to generate new cells to replace cells lost to necrosis or apoptosis and may seriously compromise the most promising and evidence-based strategies for spine regeneration, including approaches that might use stem cells and gene therapy. Therefore, all future therapeutic and regenerative strategies must first attenuate and eliminate SASP and promote a microenvironment that is more conducive to supporting endogenous or stem cell–facilitated tissue repair and regeneration.^21^, ^22^

## Molecular alterations in disc degeneration

The precise sequence of molecular events involved in the degeneration of the IVD is not clear. However, it is generally accepted that disc degeneration begins at the molecular level early in life, long before the appearance of any radiographic changes or pain symptoms. The degeneration involves a cascade of changes at the cellular and molecular level that results in degradation of the disc ECM, leading to biomechanical failure of this unique and complex structure.^23^ IVD degeneration is thought to occur where there is a loss of homeostatic balance with a predominantly catabolic metabolic profile.^24^ Once again, similar molecular mechanisms occur in joint degeneration in OA, starting with a long-lasting and asymptomatic “molecular phase,” which is followed many years later by loss of articular cartilage, evident radiographic changes, and the appearance of symptoms.^25^ Therefore, it is crucially important to understand the implications and potential impact of SASP on the efficacy of regenerative treatments and how SASP might influence the viability, metabolism, and behavior of implanted cells. It is also essential to consider the microenvironmental changes that occur with degeneration. These alterations such as decreases in oxygen, glucose, pH, and changes in osmolarity and loading are likely to influence implanted cells. These important aspects are beyond the scope of this review but they are relevant to cell-injection strategies for IVD regeneration and discussed in detail in these reviews.^22^, ^26^

## Biological and cellular approaches for intervertebral disc regeneration

Disease modifications in IVD and cartilage degeneration are extremely challenging and many existing treatments are ineffective. Progress in drug development has been painfully slow compared with other arthritic and rheumatic diseases, especially those with a more prominent inflammatory nature, including rheumatoid arthritis. Recent advances in biological therapy for disc regeneration have particularly been focusing on the nucleus pulposus (NP). This is important because the NP region of the IVD gives the tissue its unique load-bearing properties. The current approaches include using biomaterials, stem cells, and gene vectors. Stem cell–mediated cell therapy has the potential to restore the function and structure of the NP.^27^ Viral or nonviral vectors encoding functional genes may potentially generate a therapeutic effect when they are introduced into grafted cells or native cells in the NP. Biomaterial scaffolds may generate a temporally permissive microenvironment for supporting cell division and new tissue growth, allowing the remodeling of scaffolds in the regeneration process.^27^ Biomaterial scaffolds may also provide structural support for NP regeneration and serve as a carrier for stem cell and gene vector delivery, while also protecting cells and serving as a reservoir for growth factors. However, as stated earlier, as the disc degenerates, there is decreased cellularity, at least initially. Furthermore, the nutrient supply decreases as the degeneration progresses, thereby limiting cell activity and viability. Therefore, there is a close relationship between cellularity, cell activity, and nutritional support for tissue regeneration.^28^ Current biological approaches are likely to place additional demands on an already precarious nutrient supply, and this loss of nutrients associated with disc degeneration may limit the effectiveness of biological and stem cell approaches. Therefore, disc nutrition and spine regeneration are mutually interdependent.

**Fig. 2:**
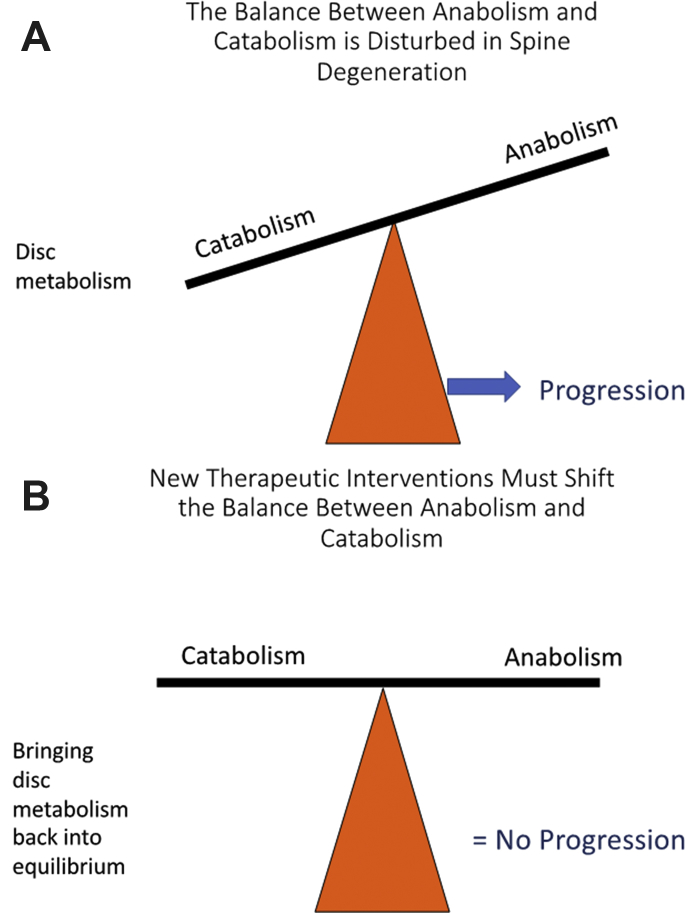
(*A*) Loss of the delicate physiologic balance between anabolism and catabolism in disc degeneration leads to impaired disc metabolism and disease progression. (*B*) Effective drugs can bring disc metabolism back into equilibrium and attenuate the rate of disease progression.

Disc degeneration essentially involves loss of homeostatic control. The balance between anabolic and catabolic activity is disturbed in disc degeneration and normal physiologic turnover of ECM macromolecules is perturbed (Fig. 2). New biological therapies must address the imbalance between catabolic and anabolic activity in order to halt disease progression. New treatments must also have the capacity to positively influence IVD metabolism.

Considering the loss of homeostatic control mechanisms, the attenuation of anabolic activity, and the elevation of catabolic processes, greater efforts must be made to stimulate anabolic activity for effective spine regeneration. This is one of the main reasons why current therapeutic strategies are now focusing on the development of cell and gene therapy and recombinant anabolic growth factors. There is significant ongoing effort in this area, especially focusing on the use of stem cells and the application of chondrocyte and stem cell–derived growth factors.

One crucial quantum leap in the development of new therapies for degenerative diseases of the joint has been the realization and acceptance that the most ideal treatments must target the affected joint through injection, directly targeting the compromised structures. The same basic concept applies to the IVD. However, many of the key researchers in this field have yet to accept that biological therapy, whether using autologous cells and stem cells, cannot be achieved with primary tissue-derived native cells. Primary and native cells do not possess the necessary regenerative attributes. Despite progress and advancements in mesenchymal stem cell (MSC) biology and the introduction of various bioactive scaffolds and growth factors in preclinical studies, current clinical trials are still at early stages with preliminary aims to evaluate safety, feasibility, and efficacy,^29^, ^30^ and this is where we must focus our efforts. Strategies that potentiate endogenous IVD disc progenitors may offer a valid alternative to the exogenous cell transplantation but new clinical trials need to be designed and carried out to make this important comparison.

Clinical trials of cell therapies, and MSCs in particular, have so far demonstrated only limited efficacy. A company called DiscGenics is about to start trials of a healthy donor NP cell therapy for IVD degeneration.^b^ Although we anxiously wait for outcomes of several ongoing stem cell trials, there are several pharmaceutical companies that have innovated in this area of cell and gene therapy by focusing efforts on developing treatments using mammalian protein production platforms, including transformed and modified cells, as well as immortalized cells that were originally developed as research tools and protein packaging cell lines for overproduction of target proteins. Biotechnology has already offered us powerful and versatile tools for the overproduction of therapeutic proteins. We need to accept the fact that new therapeutic innovations for degenerative diseases of the joints and the spine may come from other biological and biomedical disciplines, including biotechnology, protein engineering, and immunology.

## Mammalian protein production platforms

Mammalian protein production platforms are cell factories that are used for large-scale production of antibodies and therapeutic proteins.^31^ Expression of proteins in mammalian cells is an enabling technology that is vitally important for many functional studies on human and higher eukaryotic genes. Mammalian cell expression systems allow the production of proteins, especially of those of clinical relevance and human origin.^32^ Over the last few decades these platforms have evolved and had a profound impact on many areas of basic and applied research, and an increasing number of biological drugs are now recombinant mammalian proteins made using these tools.^33^ Recombinant proteins and antibodies are now produced in mammalian cell lines instead of bacterial expression systems to ensure that proper protein folding and posttranslational modifications, which are essential for full biological activity, are properly introduced in not only a eukaryotic but also a “mammalian” context.

**Fig. 3:**
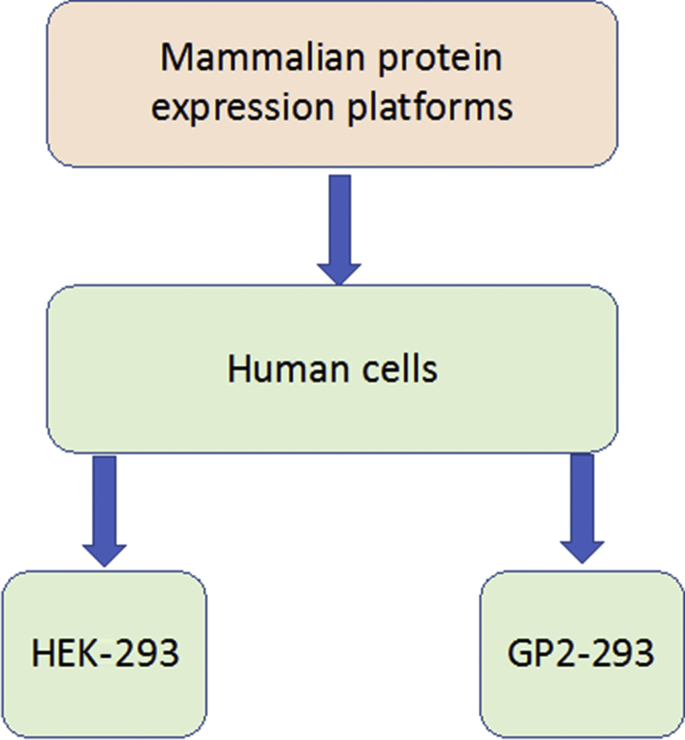
Expression systems used for human protein and glycoprotein production by biopharmaceutical industries.

Mammalian cell expression systems are the dominant tools for producing complex biotherapeutic proteins.^34^ The most commonly used mammalian cell lines found in the research and industrial therapeutic protein production settings are Chinese hamster ovary cells (CHO)^35^ and human embryonic kidney 293 cells (HEK-293).^36^ Various mammalian and nonmammalian expression systems are also being used for protein and glycoprotein production, and recent cellular engineering strategies have been developed to increase protein and glycoprotein productivity.^37^ “Omics” technologies are continually being used to improve cellular expression systems and enhance such platforms for therapeutic protein production. Fig. 3 summarizes the expression systems used for protein and glycoprotein production by industry.

(*Adapted from* Lalonde M-E, Durocher Y. Therapeutic glycoprotein production in mammalian cells. J. Biotechnol. 2017;251:129; with permission.)

Transient expression systems in mammalian cells have also become the method of choice for producing large quantities of antibodies.^38^ The ability to scale-up allows biotechnology companies to produce sufficient quantities of therapeutic antibodies and proteins for tests in preclinical studies and conducting early phase clinical trials. Without such tools, progress in biological and cellular therapy would grind to a halt.

## Platforms for overproduction of recombinant growth factors

Production of large quantities of a growth factor capable of stimulating tissue repair and new ECM synthesis will require mammalian cell models that truly mimic chondrocytes or NP cells, with phenotypic chondrocytic and NP-like properties. However, there are no such cellular tools for use in this context. However, other cellular models are available, including CHO, HEK-293 cells, and their derivatives such as GP2-293. These are immortalized cell lines that function as “cellular factories” for overproduction of proteins. GP2-293 cells are specialized protein packaging cells. These cells are specialized transfection models, protein packaging tools for overproduction of target human proteins, and are promising candidates for overproducing therapeutic proteins and growth factors that native primary cells (ie, chondrocytes, NP cells) or stem cells (ie, MSCs) cannot produce in sufficiently large quantities, either in the short term or in the long term. Although these cells cannot be used in their immortalized form for the development of clinically relevant cell therapies for the joints and the spine, they can be irradiated to obliterate their proliferation capacity so that they remain protein packaging cellular factories, but lose the ability to proliferate. Elimination of their proliferation capacity through irradiation makes the use of such cells feasible in cellular therapies, especially if the cells are to be injected into the closed microenvironment of the synovial joint or the IVD, where they will be isolated from the circulatory system. Irradiated cells will retain their capacity for protein overproduction, but they cannot divide and proliferate, which means that they will die several days after being injected into the joint or the spine. Of course there are alternative approaches to using cells. Microparticles have been developed for controlled growth differentiation factor 6 (GDF6) delivery to direct adipose stem cell–based NP regeneration. Effective encapsulation and controlled delivery of recombinant human GDF6 has been shown to maintain its activity and induced ASC differentiation to NP cells and synthesis of an NP-like matrix. Microparticles may therefore be suitable for controlled growth factor release in regenerative strategies for treatment of IVD degeneration.^39^ However, transformed cells and protein production platforms have the potential for controlled and sustained growth factor synthesis and release over a period of days.

## Production of transforming growth factor β1 (TGF-β1) by protein packaging cells GP2-293 in the Kolon TissueGene’s cell and gene therapy product—Invossa

**Fig. 4:**
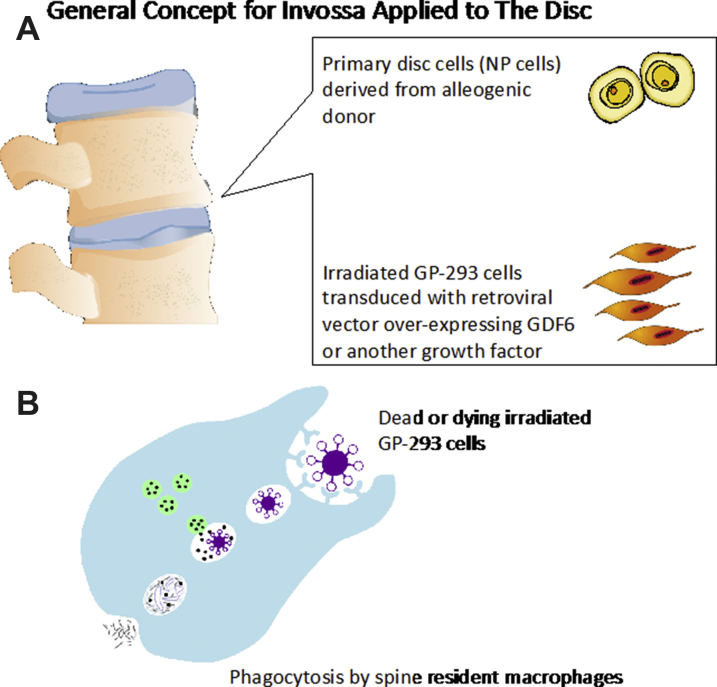
(*A*) The intraarticular injection concept for Invossa, originally developed as a novel cell and gene therapy targeting knee OA, can be modified and repurposed for IVD regeneration. In this concept injection of primary NP cells or stem cells and irradiated GP2-293 overproduce a suitable growth factor, such as GDF6. This is the biological growth factor that is thought to promote the anabolic repair and regeneration of IVD. Alternatively GDF6 could be used with any other growth factor or combination of growth factors as the field of IVD regeneration progresses. (*B*) Phagocytosis and destruction of dead GP2-293 or their cellular debris by spine resident macrophages.

Invossa^c^ is a unique first-in-class cell and gene therapy targeting knee OA through a single intraarticular injection of joint-derived chondrocytes, irradiated GP2-293, and, most importantly, the biological growth factors that they overproduce to possibly promote anabolic repair and regeneration in the diseased joint as a future possibility in the treatment of OA. The same scientific principle may be applied to the degenerated disc (Fig. 4A), where TGF-β could be replaced by a more appropriate growth factor such as GDF6.^40^ Therefore, a more suitable growth factor such as GDF6 can be overproduced instead.

If regenerating the NP region is desired, native patient-derived NP cells will not have the capacity to overproduce an anabolic growth factor in sufficiently high quantities for successful cellular therapy and regenerative applications. Human GP2-293 cells are one of the key components of Invossa and carry out the vital function of overproducing the crucially important growth factor; hence they offer an attractive alternative for IVD regeneration strategies. GP2-293 cells have been used throughout the whole developmental process for Invossa, from the first production of the Master Cell Bank to the next step, which is the development of the working cell bank and the final product formulation. As mentioned earlier, GP2-293 is an HEK-293–based retroviral packaging cell line used for large-scale protein production. It is a cellular platform for overproduction of therapeutically relevant human proteins. This is the first time that such a human protein production platform has been used in the context of OA treatment and cartilage regeneration. It is conceivable that the same approach may work for the IVD, whether with TGFβ or GDF6 or an alternative growth factor, as these cells can function as a protein-producing tool and “cellular factory” for therapeutic growth factors that can target degenerative pathways in IVD.

## Safety of GP2-293 cells in Invossa

Transduced and irradiated GP2-293 cells may be transformed triploid cells but they have lost their capacity for proliferation through irradiation. Therefore, the GP2-293 cells in Invossa cannot survive and proliferate in the joint or in the spine. It is envisaged that these cells will simply carry out their transient function as radiation inactivated transfection models, protein packaging tools, and “cellular factories” for overproduction of therapeutic growth factors such as TGF-β1. Therefore, the cells cannot survive for more than a very short period of days after being injected into the joint or the spine. After the cells carry out their TGF-β1 production duties, they will die and their remains will be cleared by joint resident inflammatory macrophages through the process of phagocytosis (Fig. 4B).

The scientific basis for the use of mammalian cell transfection models in new cell-based therapies for the spine is clear in the development of Invossa. There is a well-established literature on the use of HEK-293 cells as a transfection model and cell culture model for protein production in a research setting but there is potential to extend this concept to clinically relevant biological therapies. The efficacy and safety of HEK-293 cells and their derivatives in cell therapy has not been extensively investigated but the prospects for future use of transfection tools in regenerative medicine is very positive, especially because native and untransformed cells do not have the appropriate regenerative capacity.

## Summary

Cell and gene therapy for degenerative diseases of the joint and spine is a promising area of research with significant potential for clinical development. However, there are currently no effective treatments for spine degeneration. The significant recent advances in the field of biotechnology are likely to have a positive impact on tissue engineering and regenerative treatments for the spine. We need to accept the harsh reality that primary, aged, and senescent cells are unlikely to possess robust regenerative properties. Regenerative medicine and tissue engineering strategies for the spine should consider the use of stem cells combined with mammalian protein production platforms to drive the production of therapeutic proteins and growth factors.

## Financial support and sponsorship

Funding for S.M. Richardson is acknowledged from the Biotechnology and Biological Sciences Research Council; the Engineering and Physical Sciences Research Council; and the Medical Research Council [grant number MR/K026682/1] via the UK Regenerative Medicine Platform Hubs “Acellular Approaches for Therapeutic Delivery”, as well as the Medical Research Council via a Confidence-in-Concept 2014 award to The University of Manchester (MC_PC_14112 v.2). A. Mobasheri has received funding from the following sources: The European Commission Framework 7 programme (EU FP7; HEALTH.2012.2.4.5-2, project number 305815; Novel Diagnostics and Biomarkers for Early Identification of Chronic Inflammatory Joint Diseases). The Innovative Medicines Initiative Joint Undertaking under grant agreement No. 115770, resources of which are composed of financial contribution from the European Union’s Seventh Framework programme (FP7/2007-2013) and EFPIA companies’ in-kind contribution. The author also wishes to acknowledge funding from the European Commission through a Marie Curie Intra-European Fellowship for Career Development grant (project number 625746; acronym: CHONDRION; FP7-PEOPLE-2013-IEF). A. Mobasheri also wishes to acknowledge financial support from the European Structural and Social Funds (ES Struktūrinės Paramos) through the Research Council of Lithuania (Lietuvos Mokslo Taryba) according to the activity ‘Improvement of researchers’ qualification by implementing world-class R&D projects’ of Measure No. 09.3.3-LMT-K-712 (grant application code: 09.3.3-LMT-K-712-01-0157, agreement No. DOTSUT-215) and the new funding programme: Attracting Foreign Researchers for Research Implementation (2018-2022) [grant No 0.2.2-LMTK-718-02-0022]. The author has received payments from King Abdulaziz University, Jeddah, Kingdom of Saudi Arabia. The author also declares that he has consulted for the following companies in the last three years: Abbvie, Aché Laboratórios Farmacêuticos S.A., AlphaSights, Galapagos, Guidepoint Global, Kolon TissueGene, Pfizer Consumer Health (PCH), Servier, Bioiberica S.A. and Science Branding Communications.

## Footnotes

a https://www.who.int/bulletin/volumes/81/9/Ehrlich.pdf.

b https://www.prnewswire.com/news-releases/discgenics-announces-first-patient-treated-in-us-clinical-trial-of-idct-for-degenerative-disc-disease-300636859.html.

c Kolon TissueGene (KTG) is a US based company and they have TG-C in clinical trials. Kolon Life Sciences (KLS) is based in Korea and they have the marketed product Invossa-K. If approved in the United States, KTG will have Invossa in the United States.
